# Características Associadas à Fibrilação Atrial Prevalente e Perfil de Risco para Fibrilação Atrial Incidente em uma População Idosa do ELSA-Brasil

**DOI:** 10.36660/abc.20240487

**Published:** 2025-01-30

**Authors:** Bernardo Boccalon, Murilo Foppa, Luisa C.C Brant, Marcelo M. Pinto-Filho, Antonio L. Ribeiro, Bruce B. Duncan, Angela Barreto Santiago Santos

**Affiliations:** 1 Universidade Federal do Rio Grande do Sul Porto Alegre RS Brasil Universidade Federal do Rio Grande do Sul, Porto Alegre, RS – Brasil; 2 Hospital de Clinicas de Porto Alegre Porto Alegre RS Brasil Hospital de Clinicas de Porto Alegre, Porto Alegre, RS – Brasil; 3 Universidade Federal de Minas Gerais Belo Horizonte MG Brasil Universidade Federal de Minas Gerais, Belo Horizonte, MG – Brasil

**Keywords:** Fibrilação Atrial, Idoso, Fatores de Risco, Epidemiologia

## Abstract

**Fundamento:**

A fibrilação atrial (FA) é uma arritmia que causa sintomas significativos e aumenta o risco de complicações.

**Objetivos:**

Avaliar a associação de parâmetros clínicos, eletrocardiográficos e ecocardiográficos com fibrilação ou flutter atrial (FFA) prevalente e avaliar o perfil de risco para FFA incidente utilizando os escores de predição de FA CHARGE-AF e EHR em uma população idosa de um país em desenvolvimento.

**Métodos:**

Incluímos todos os participantes do ELSA-Brasil com 60 anos ou mais cujo diagnóstico de FFA pôde ser definido por autorrelato ou eletrocardiograma e que tiveram ecocardiografia realizada na linha de base do estudo. Para a análise estatística, foram considerados estatisticamente significativos os resultados com valores de p < 0,05.

**Resultados:**

Dos 2.088 participantes (65 ± 4,1 anos; 53% mulheres), 88 (4,2%) tinham FFA. Aqueles com FFA eram mais velhos e tinham maiores taxas de insuficiência cardíaca (IC), infarto do miocárdio prévio, bloqueio de ramo esquerdo (BRE), intervalo QT prolongado, extrassístoles supraventriculares e bradicardia sinusal. Esses pacientes também apresentavam maiores dimensões do átrio esquerdo e do ventrículo esquerdo e menor fração de ejeção do ventrículo esquerdo (FEVE). A análise multivariada mostrou que insuficiência cardíaca, BRE, átrio esquerdo maior e FEVE menor estavam independentemente associados com FFA. O risco de 5 anos para FFA incidente foi baixo (< 2,5%) em 63% e alto (> 5%) em 12% dos indivíduos de acordo com o escore CHARGE-AF, e baixo em 67% e alto em 13% de acordo com o EHR.

**Conclusão:**

A FFA foi encontrada em 4,2% da presente coorte brasileira idosa. A FFA foi associada ao histórico de insuficiência cardíaca, BRE, dilatação do átrio esquerdo e FEVE reduzida. Adicionalmente, 12% a 13% dos pacientes em ritmo sinusal estavam em alto risco para FFA. O monitoramento de parâmetros clínicos, eletrocardiográficos e ecocardiográficos pode auxiliar na identificação precoce de indivíduos de alto risco.

## Graphical abstract

**Figura Central:**
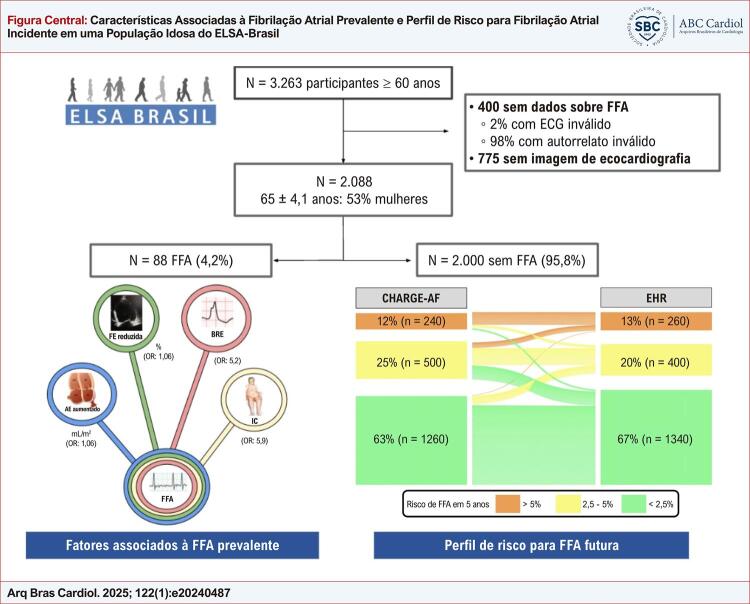
Características Associadas à Fibrilação Atrial Prevalente e Perfil de Risco para Fibrilação Atrial Incidente em uma População Idosa do ELSA-Brasil

Figura Central – Variáveis associadas à fibrilação atrial prevalente e perfil de risco para fibrilação atrial futura na população idosa do ELSA-Brasil. AE: átrio esquerdo; BRE: bloqueio de ramo esquerdo; FE: fração de ejeção; FFA: fibrilação ou flutter atrial; IC: insuficiência cardíaca; OR: odds ratio.

## Introdução

A fibrilação atrial (FA) é a arritmia cardíaca sustentada mais comum e pode levar a sintomas limitantes e aumento do risco de acidente vascular cerebral, infarto do miocárdio (IM), insuficiência cardíaca (IC) e mortalidade por todas as causas.^1 - 3^ A prevalência de FA varia entre diferentes populações, atingindo 1% a 2% da população geral, e aumenta com a idade, particularmente em indivíduos com mais de 60 anos.^4^ No entanto, a disponibilidade limitada de dados sobre a prevalência de FA em países de baixa e média renda representa um desafio significativo aos esforços globais de produzir conhecimento sobre a FA.^5^

A fibrilação ou flutter atrial (FFA) é um problema crescente no Brasil devido à sua transição epidemiológica, com o envelhecimento acelerado da nossa população e o aumento das doenças cardiovasculares. No ELSA-Brasil, uma grande coorte multicêntrica de pacientes entre 35 a 74 anos no Brasil, a prevalência de FFA foi de 2,5%, aumentando progressivamente com o envelhecimento (1,2% para pacientes < 45 anos e 5,4% para aqueles > 64 anos).^6^ Outro estudo brasileiro que incluiu 1.524 indivíduos com 65 anos ou mais encontrou uma prevalência de 2,4% dessa arritmia.^7^

A presença de fatores de risco ao longo de décadas pode justificar o aumento da incidência de FFA com o envelhecimento, tornando os idosos mais suscetíveis ao desenvolvimento de arritmias.^8^ Ademais, em idosos, a FFA está associada a um risco significativamente maior de complicações, bem como a uma chance maior de evoluir para FFA permanente, em comparação aos pacientes jovens.^9^ Considerando que aproximadamente 40% dos indivíduos com FFA são clinicamente assintomáticos, o diagnóstico dessa arritmia pode ocorrer somente após o desenvolvimento de suas consequências.^10^

A identificação dos fatores de risco associados à FFA, bem como dos indivíduos que apresentam escores de risco mais altos para desenvolver essa arritmia, pode auxiliar no monitoramento de novos casos ou retardar a progressão da doença, permitindo tratamento precoce e redução de complicações. Assim, visamos avaliar a associação de parâmetros clínicos, eletrocardiográficos e ecocardiográficos com FFA prevalente em adultos com 60 anos ou mais em uma grande coorte brasileira e descrever o perfil de risco de novos casos de FFA nessa população.

## Métodos

### População do estudo

**Figura 1:**
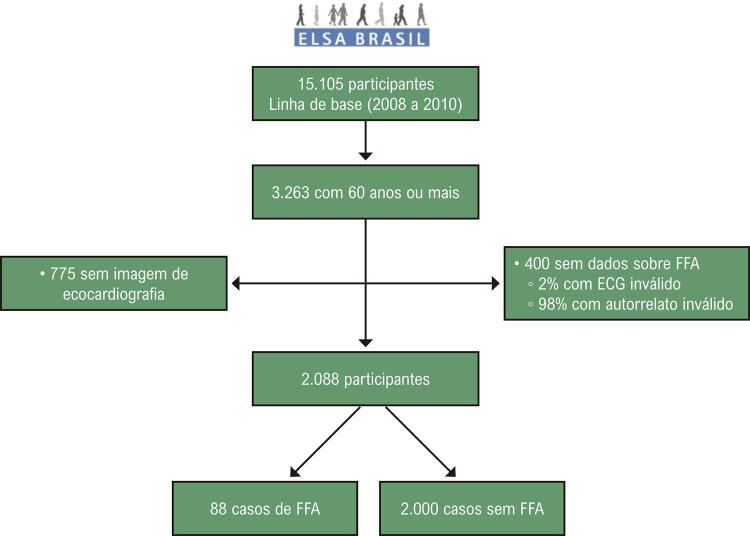
– Fluxograma do estudo.

O ELSA-Brasil é um estudo de coorte prospectivo projetado para investigar doenças cardiovasculares e diabetes em 15.105 homens e mulheres, servidores públicos de universidades ou instituições de pesquisa em 6 cidades do Brasil. Todos os funcionários ativos ou aposentados com idade entre 35 e 74 anos foram elegíveis para o estudo. Incluímos todos os participantes com 60 anos ou mais cujo diagnóstico de FFA pôde ser avaliado e que tinham ecocardiografia na linha de base do estudo. Da amostra inicial com participantes ≥ 60 anos (n = 3.263), 400 participantes foram excluídos devido a dados indisponíveis sobre FFA (8 devido a eletrocardiograma inválido e 392 devido a informações ausentes em relação a FFA anterior). Dos 2.863 participantes restantes, 2.088 tinham imagens ecocardiográficas disponíveis para análise ( Figura 1 ).

Os detalhes do estudo, incluindo o desenho, critérios de elegibilidade, fontes, métodos de recrutamento e medidas obtidas foram descritos em outras publicações.^11 - 13^ O protocolo do estudo foi aprovado pelo Comitê de Revisão Institucional de cada centro participante, e o consentimento informado por escrito foi fornecido por todos os participantes. A presente investigação foi um estudo transversal do ELSA-Brasil durante a primeira consulta (agosto de 2008 a dezembro de 2010).

### Diagnóstico de fibrilação ou flutter atrial

O presente estudo definiu o diagnóstico de FFA na linha de base se o participante (a) tinha um registro de eletrocardiograma com FFA no ELSA-Brasil na avaliação inicial (2008 a 2010) (n = 24) ou (b) indicou que tinha um diagnóstico de FA em uma idade mais jovem do que a da inscrição no ELSA-Brasil pelo questionário aplicado 4 anos após a inscrição (n = 64). Entre 2012 e 2014, os participantes foram convidados a passar por uma reavaliação no local, que incluiu novos questionários. Nessa reavaliação, foi feita a seguinte pergunta: “Algum médico já disse que o senhor/a senhora tem/teve fibrilação atrial?” Os participantes que responderam “sim” a essa pergunta foram questionados: “Quantos anos o senhor/a senhora tinha na primeira vez que um médico lhe disse que tem/teve fibrilação atrial?”^6^

### Variáveis clínicas

A escolha das variáveis clínicas foi baseada nas variáveis incluídas nas fórmulas de estimativa de risco CHARGE-AF^14^ e EHR.^15^ Variáveis demográficas e clínicas foram obtidas na visita inicial de acordo com protocolos padronizados. A hipertensão foi definida como pressão arterial sistólica ≥ 140 mmHg ou pressão arterial diastólica ≥ 90 mmHg, aferidas na visita clínica do estudo, ou ainda como tratamento com medicação anti-hipertensiva durante as últimas 2 semanas. Os participantes foram classificados como portadores de diabetes se relataram diagnóstico prévio de diabetes, estavam tomando medicamentos para diabetes ou apresentaram um dos seguintes resultados nos exames laboratoriais: glicemia de jejum ≥ 126 mg/dl, glicemia de 2 horas ≥ 200 mg/dl ou HbA1C ≥ 6,5%. A dislipidemia foi definida como uso de medicamentos hipolipemiantes ou qualquer um dos seguintes resultados: nível de colesterol LDL ≥ 130 mg/dl, colesterol total > 200 mg/dl, HDL-C < 40 mg/dl em homens ou < 50 mg/dl em mulheres, ou triglicerídeos > 150 mg/dl. A taxa de filtração glomerular (TFG) foi estimada usando a fórmula da Chronic Kidney Disease Epidemiology Collaboration (CKD-EPI), e a doença renal crônica foi definida como TFG < 60 ml/min.

As condições médicas prévias, incluindo o diagnóstico de IC, IM e acidente vascular cerebral foram obtidas por meio de entrevistas e questionários padronizados do estudo. Os participantes que declararam ter fumado pelo menos 100 cigarros ao longo da vida e que continuavam fumando foram considerados fumantes ativos.^12 , 13^ A classificação racial foi baseada em relatos autodeclarados usando categorias do Censo Demográfico brasileiro conduzido pelo Instituto Brasileiro de Geografia e Estatística.^16^

Adicionalmente, foram calculados 4 escores de risco com o objetivo de estimar o risco de eventos embólicos (CHADsVASc), risco cardiovascular (Doença Cardiovascular Aterosclerótica [ASCVD]) e a incidência de FFA em 5 anos (EHR e CHARGE-AF) e, subsequentemente, caracterizar nossa população identificando os pacientes com doença mais grave.

### Variáveis eletrocardiográficas

As variáveis eletrocardiográficas foram obtidas em uma consulta na linha de base. O procedimento para aquisição e leitura de eletrocardiogramas foi detalhado em uma publicação anterior^11^ e inclui procedimentos de garantia de qualidade estabelecidos. Os eletrocardiogramas foram realizados em cada centro de investigação usando o dispositivo Burdick Atria 6100, com calibração de 10 mm/mV e velocidade de 25 mm/segundo. Os exames foram transmitidos para o centro de leitura eletronicamente e armazenados em um banco de dados digital para subsequente leitura automatizada pelo Sistema de Glasgow^17^ e codificação pelo Código de Minnesota.^18^ Todos os eletrocardiogramas com FFA foram verificados manualmente por um médico.

### Análises ecocardiográficas

Todas as imagens ecocardiográficas foram realizadas na primeira visita por ecocardiografistas treinados, usando equipamento idêntico (Aplio XG, Toshiba Corporation, Toshigi, Japão), com um transdutor setorial de 2,5 MHz. Sequências de 3 ciclos cardíacos foram selecionadas em cada janela ecocardiográfica, registradas em formato digital e transferidas para o centro de leitura ecocardiográfica do ELSA-Brasil, juntamente com um formulário digital com qualidade de imagem e achados preliminares preenchidos pelo ecocardiografista. Todos os estudos foram analisados cegamente para outros dados dos participantes em uma estação específica de trabalho offline (ComPACS Review Station 10.5, Medimatic Solutions Srl, Itália). Todas as medições foram feitas em triplicado seguindo as recomendações da American Society of Echocardiography^19^ e incluíram diâmetros do ventrículo esquerdo (VE), espessura da parede do VE, massa do VE, fração de ejeção do ventrículo esquerdo (FEVE) e volume do átrio esquerdo (AE).

### Modelos de previsão de risco de FFA

Utilizamos 2 modelos de previsão de risco publicados para FFA: o CHARGE-AF e o registro eletrônico de saúde para FA (EHR), que estimam o risco cumulativo de FFA incidente em 5 anos de acordo com as seguintes 3 categorias de risco: baixo (< 2,5%), intermediário (2,5% a 5%) e alto (> 5%).^14 , 15^ O modelo previsto CHARGE-AF para FFA considerou fatores como idade, raça, altura, peso, pressão arterial sistólica e diastólica, tabagismo atual, uso de medicamentos anti-hipertensivos, diabetes e histórico de IM agudo e IC.^14^ O escore EHR foi desenvolvido pela análise de dados de 412.085 indivíduos. O modelo incorporou variáveis como sexo, idade, raça, tabagismo, altura, peso, pressão arterial diastólica, hipertensão, hiperlipidemia, IC, doença cardíaca coronária, doença valvar, histórico de acidente vascular cerebral, doença arterial periférica, doença renal crônica e hipotireoidismo.^15^

### Declaração ética

O presente estudo foi realizado de acordo com os princípios da Declaração de Helsinque. Por se tratar de um estudo multicêntrico, o protocolo de pesquisa do ELSA-Brasil foi aprovado não apenas pelo comitê de ética de cada instituição, mas também pelo Comitê Nacional de Ética em Pesquisa. Foi obtido o consentimento informado de todos os sujeitos envolvidos no estudo.

### Análise estatística

Todos os dados com distribuição normal foram descritos como média e desvio padrão (dados contínuos) ou como contagem e proporção (dados categóricos). As variáveis contínuas foram comparadas usando um teste t bilateral com variância desigual e as variáveis categóricas foram comparadas usando testes qui-quadrado. Os dados contínuos com distribuição não normal foram descritos como mediana e intervalo interquartil e analisados usando o teste U de Mann-Whitney. Foi usado o teste de Shapiro-Wilk para avaliar a normalidade dos dados.

As variáveis potencialmente associadas à FFA foram analisadas usando regressão logística univariada. Um modelo logístico multivariável foi usado para identificar variáveis clinicamente relevantes e não concorrentes, usando apenas parâmetros com significância estatística na regressão logística univariada. Os modelos de predição de risco para FFA foram calculados e foi utilizado o diagrama de Sankey^20^ para comparar a sobreposição dessas duas classificações. Todos os testes foram bilaterais, e foram considerados estatisticamente significativos valores de p < 0,05. As análises estatísticas foram realizadas com STATA 14.0 (Stata Corp, College Station, TX, EUA).

## Resultados

Houve 88 participantes (4,2%) com FFA entre os 2.088 participantes incluídos no presente estudo (65 ± 4,1 anos, 53% mulheres e 57% autodeclarados brancos). Os participantes excluídos da análise, devido à falta de informações sobre FFA ou ecocardiografia, eram mais obesos com maior prevalência de diabetes, doença renal crônica e tabagismo ativo, conforme mostrado na Tabela Suplementar 1.

Conforme mostrado na Tabela 1 , os participantes com FFA eram mais velhos e tinham um pior perfil de risco cardiovascular. Eles apresentaram maior prevalência de IC e IM anteriores, maior risco de eventos cardiovasculares em 10 anos de acordo com o escore ASCVD e maior risco de eventos embólicos relacionados à FFA, de acordo com o escore CHADsVASc calculado. Não foram verificadas diferenças relacionadas ao sexo, presença de hipertensão, obesidade e histórico de acidente vascular cerebral entre os grupos. Anormalidades eletrocardiográficas, como bloqueio do ramo esquerdo (BRE) completo, intervalo QT prolongado, extrassístoles supraventriculares e bradicardia sinusal, foram mais prevalentes em indivíduos com FFA ( Tabela 2 ). Os parâmetros ecocardiográficos em participantes com FFA demonstraram maiores dimensões do AE (parâmetros lineares e volumétricos) e do VE, menor FEVE e doença valvar esquerda moderada a grave mais prevalente em comparação com os casos sem FFA ( Tabela 3 ). No material suplementar, mostramos a análise restrita aos 24 participantes que tinham FFA no ECG basal (Tabelas Suplementares 2, 3 e 4).

**Tabela 1:** – Características clínicas e demográficas da amostra estudada de participantes com 60 anos ou mais na linha de base do Estudo ELSA-Brasil

	Todos os participantes (n=2.088)	FFA (n=88)	Sem FFA (n=2.000)	Valor p
Idade, anos	65 ± 4,1	66,8 ± 4,4	65 ± 4,0	0,002
Mulheres, n (%)	1.111 (53)	43 (50)	1.068 (53,4)	0,40
Raça branca, n (%)	1.179 (56,9)	54 (61,3)	1.125 (56,7)	0,39
Peso, kg	72,04 ± 14,0	72,84 ± 14,97	72,01 ± 13,97	0,60
Índice de massa corporal, kg/m^2^	27,09 ± 4,49	26,9 ± 4,44	27,09 ± 4,49	0,69
Obesidade, n (%)	468 (22,4)	21 (23,86)	447 (22,35)	0,73
Hipertensão, n (%)	1.211 (58,1)	56 (63,63)	1155 (57,8)	0,28
Pressão arterial sistólica, mmHg	129,1 ± 19,02	127,5 ± 15,98	129,2 ± 19,14	0,35
Pressão arterial diastólica, mmHg	76,51 ± 10,71	75,26 ± 10,03	76,57 ± 10,74	0,23
Diabetes mellitus, n (%)	549 (26,3)	29 (32,95)	520 (26,01)	0,14
Insuficiência cardíaca, n (%)	77 (3,69)	14 (16,09)	63 (3,15)	<0,001
Infarto agudo do miocárdio, n (%)	90 (4,3)	13 (14,77)	77 (3,85)	<0,001
Acidente vascular cerebral, n (%)	53 (2,54)	4 (4,55)	49 (2,45)	0,22
Tabagismo ativo, n (%)	171 (8,19)	10 (11,36)	161 (8,05)	0,26
Dislipidemia, n (%)	1.805 (86,8)	78 (88,6)	1.727 (86,7)	0,60
Doença renal crônica, n (%)	157 (7,5)	11 (12,5)	146 (7,3)	0,07
Hipotireoidismo, n (%)	219 (10,5)	12 (13,6)	207 (10,4)	0,33
Escore CHARGE-AF				<0,001
Risco baixo, n (%)	1.298 (62,2)	38 (43,2)	1.260 (63)	
Risco intermediário, n (%)	521 (24,9)	21 (23,9)	500 (25)	
Risco alto, n (%)	269 (12,9)	29 (32,9)	240 (12)	
Escore EHR				<0,001
Risco baixo, n (%)	1.384 (66,3)	44 (50)	1.340 (67)	
Risco intermediário, n (%)	419 (20,1)	19 (21,6)	400 (20)	
Risco alto, n (%)	285 (13,6)	25 (28,4)	260 (13)	
CHADsVASc	2,06 ± 1,20	2,54 ± 1,27	2,04 ± 1,20	<0,001
ASCVD 2013, %	11,4 (6,2 - 19,8)	13,8 (8,44 - 22,3)	11,4 (6 - 19,7)	0,012

Os números representam média ± desvio padrão ou mediana e intervalo interquartil para variáveis contínuas e n (%) para variáveis categóricas. ASCVD: Estimativa de Risco de Doença Cardiovascular Aterosclerótica em 10 anos; FFA: fibrilação ou flutter atrial.

**Tabela 2:** – Parâmetros eletrocardiográficos da amostra estudada de participantes com 60 anos ou mais na linha de base do Estudo ELSA-Brasil

	Todos os participantes (n=2.064)	FFA (n=64)	Sem FFA (n=2,000)	Valor p
Duração da onda P, ms	110,05 ± 14,7	110,62 ± 16,5^*^	110,03 ± 14,71	0,79
Intervalo PR longo,^#^ n (%)	46 (2,22)	3 (3,45)^*^	43 (2,17)	0,42
Anormalidades maiores da onda Q, n (%)	87 (4,2)	3 (3,4)	84 (4,2)	0,71
Bloqueio completo do ramo direito, n (%)	80 (3,8)	4 (4,6)	76 (3,8)	0,71
Bloqueio completo do ramo esquerdo, n (%)	22 (1,06)	4 (4,6)	18 (0,91)	0,001
HVE com alterações ST-T, n (%)	14 (0,68)	0 (0)	14 (0,71)	0,43
QT longo, n (%)	46 (2,2)	7 (7,95)	39 (1,96)	<0,001
Desvio do eixo esquerdo, n (%)	125 (6,04)	6 (6,9)	119 (6)	0,73
Desvio do eixo direito, n (%)	1 (0,05)	0 (0)	1 (0,05)	0,83
Extrassístoles supraventriculares, n (%)	27 (1,3)	4 (4,6)^*^	23 (1,16)	0,006
Extrassístoles ventriculares, n (%)	9 (0,43)	1 (1,15)	8 (0,40)	0,30
Bradicardia sinusal, n (%)	68 (3,27)	10 (11,36)^*^	58 (2,91)	<0,001
Baixa voltagem do QRS, n (%)	40 (1,93)	3 (3,45)	37 (1,86)	0,29

Os números representam a média ± desvio padrão e n (%) para variáveis categóricas. *Dados de participantes que estavam em ritmo sinusal durante o eletrocardiograma basal, mas foram definidos como tendo FFA por autorrelato. #Considerado acima de 200 ms. FFA: fibrilação ou flutter atrial; HVE: hipertrofia ventricular esquerda.

**Tabela 3:** – Parâmetros ecocardiográficos da amostra estudada de participantes com 60 anos ou mais na linha de base do Estudo ELSA-Brasil

	Todos os participantes (n=2.088)	FFA (n=88)	Sem FFA (n=2.000)	Valor p
Diâmetro do átrio esquerdo, cm	3,61 ± 0,51	3,91 ± 0,73	3,60 ± 0,49	<0,001
Índice de volume atrial esquerdo, mL/m^2^	27,3 ± 8,44	33,3 ± 14,8	27 ± 7,92	<0,001
Diâmetro diastólico final do VE, cm	4,48 ± 0,50	4,63 ± 0,6	4,47 ± 0,4	0,02
Diâmetro sistólico final do VE, cm	2,81 ± 0,47	3,07 ± 0,7	2,79 ± 0,45	0,001
Espessura do septo, cm	1,03 ± 0,18	1,02 ± 0,18	1,03 ± 0,18	0,84
Espessura da parede posterior, cm	0,92 ± 0,14	0,95 ± 0,13	0,92 ± 0,14	0,06
Fração de ejeção do VE, %	67,3 ± 6,6	63,6 ± 9,1	67,4 ± 6,42	<0,001
Massa do VE/ASC, g/m^2^	85 ± 21,3	89,6 ± 22,6	84,8 ± 21,9	0,06
Doença valvar esquerda moderada a grave, n (%)	42 (2)	6 (6,8)	36 (1,8)	0,001
Padrões geométricos do VE, n (%)				0,15
Normal	923 (49)	37 (46,7)	886 (49,8)	
Remodelação concêntrica	655 (35)	24 (30,4)	632 (35,5)	
Hipertrofia concêntrica	157 (9)	12 (15,2)	145 (8,2)	
Hipertrofia excêntrica	122 (7)	6 (7,6)	116 (6,5)	
Relação E/e'	8,7 ± 2,5	8,8 ± 2,3	8,7 ± 2,5	0,72

Os números representam a média ± desvio padrão e n (%) para variáveis categóricas. ASC: área de superfície corporal; FFA: fibrilação ou flutter atrial; VE: ventrículo esquerdo.

As *odds ratios* de cada variável para a presença de FFA estão resumidas na Tabela 4 . Na regressão logística multivariável, histórico de IC, presença de BRE, índice de volume atrial esquerdo e FEVE foram independentemente associados com FFA prevalente.

**Tabela 4:** – Fatores de risco associados à presença de fibrilação ou flutter atrial na linha de base do Estudo ELSA-Brasil na amostra estudada de participantes com 60 anos ou mais

Variáveis	Regressão logística univariada			Regressão logística multivariada		
	Odds ratio	IC 95%	Valor p	Odds ratio	IC 95%	Valor p
Idade, anos	1,1	1,05-1,16	<0,001			
Mulheres	0,87	0,56-1,34	0,53			
Índice de massa corporal, kg/m^2^	0,99	0,94-1,03	0,69			
Insuficiência cardíaca	5,9	3,15 - 11	<0,001	2,56	1,09-6,01	0,03
Infarto do miocárdio anterior	4,32	2,3- 8,12	<0,001			
Hipertensão	1,27	0,81-1,98	0,28			
Diabetes mellitus	1,39	0,88-2,20	0,14			
Acidente vascular cerebral	1,89	0,66-5,37	0,22			
Bloqueio completo do ramo esquerdo	5,26	1,74-15,9	0,003	4,97	1,23-20	0,024
QT longo	4,33	1,88-9,98	0,001			
Extrassístoles supraventriculares	4,1	1,38-12,1	0,011			
Bradicardia sinusal	4,27	2,1-8,69	<0,001			
Diâmetro diastólico final do VE (cm)	1,75	1,16-2,65	0,007			
Diâmetro sistólico final do VE (cm)	2,38	1,65-3,42	<0,001			
Índice de volume atrial esquerdo (mL/m^2^)	1,06	1,04-1,08	<0,001	1,04	1,02-1,07	<0,001
Massa do VE/ASC (g/m^2^)	1,0	0,99-1,01	0,05			
Fração de ejeção do VE (%)	0,94	0,90-0,96	<0,001	0,95	0,91-0,97	0,001
Doença valvar esquerda moderada a grave	3,99	1,63-9,74	0,002			

ASC: área de superfície corporal; FFA: fibrilação ou flutter atrial; IC: intervalo de confiança; VE: ventrículo esquerdo.

**Figura 2:**
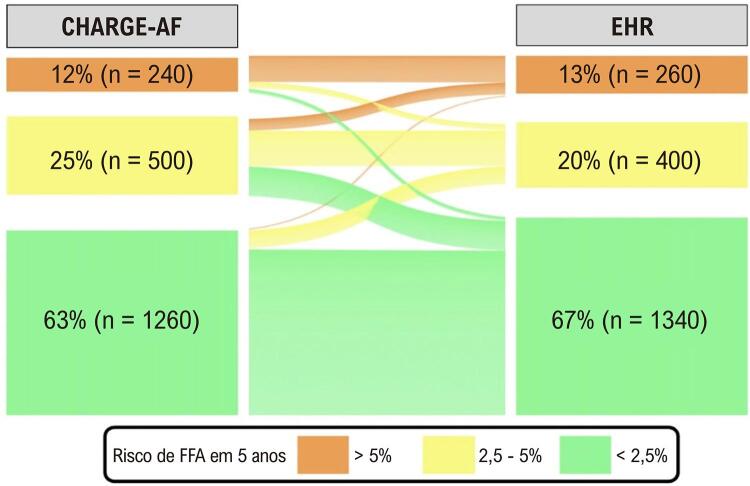
– Interseção entre o perfil de previsão de risco de FFA em 5 anos: CHARGE-AF e EHR. FFA: fibrilação ou flutter atrial.

Ambos os modelos de risco mostraram que a maioria dos 2.000 pacientes em ritmo sinusal na linha de base do estudo apresentavam baixo risco de desenvolver FFA. De acordo com o CHARGE-AF, 63% dos indivíduos foram classificados como de baixo risco (< 2,5%) e 12% como de alto risco, com um risco de FFA maior que 5% em de 5 anos. Da mesma forma, de acordo com o EHR, 67% dos participantes foram classificados como de baixo risco e 13% foram considerados de alto risco. Na Figura 2 , demonstramos a distribuição dos escores entre indivíduos em ritmo sinusal e a sobreposição elevada entre os escores, com 71% deles classificados como risco semelhante em ambos os escores. A mudança mais significativa na categoria de risco ocorreu entre os pacientes classificados como risco intermediário de acordo com o escore CHARGE-AF, que foram reclassificados como de baixo risco pelo escore EHR.

## Discussão

No presente estudo, demonstramos que o histórico médico de IC, BRE, tamanho do AE e FEVE estavam independentemente associados à FFA prevalente em uma população idosa brasileira. Adicionalmente, verificamos que 12% a 13% daqueles em ritmo sinusal apresentavam alto risco de desenvolver FFA nos 5 anos seguintes, independentemente do escore de risco usado.

A idade avançada tem sido consistentemente identificada como um dos principais fatores de risco para FFA,^3 , 21 - 23^ apontando para o papel potencial da senescência celular na fisiopatologia da FA,^24^ e mudanças recentes na expectativa de vida podem potencialmente aumentar a prevalência dessa arritmia. Semelhante a um estudo anterior do ELSA-Brasil,^6^ o estudo de Rotterdam (n = 6.808) revelou que a prevalência de FFA foi de 9% em indivíduos entre 75 e 79 anos, aumentando significativamente para 17,8% em indivíduos com 85 anos ou mais.^25^ Em nosso estudo, não verificamos que a idade foi um fator independente para a prevalência de FFA, o que pode ser explicado pelo fato de termos restringido nossa amostra a uma faixa limitada de 60 a 74 anos. Além disso, as particularidades de nossa amostra, composta principalmente por servidores públicos ativos, podem limitar o achado da associação entre FFA e outros fatores estabelecidos para FFA. O acidente vascular cerebral pode ter sido sub-representado devido às limitações impostas por essa condição à participação em atividades de trabalho e, consequentemente, em pesquisas. Ademais, a transição epidemiológica observada na população estudada, na qual a obesidade historicamente não foi um fator de risco prevalente, pode explicar a ausência desse fator de risco em idosos. Por fim, a alta prevalência de hipertensão em ambos os grupos pode ter anulado as diferenças relacionadas a essa situação específica.

Verificamos que um histórico de IC estava associado à FFA prevalente. Anteriormente, Benjamin e coautores demonstraram que a presença de IC aumentou em 6 vezes o risco de desenvolver FA em um longo acompanhamento do Framingham Heart Study (38 anos).^26^ Essa ligação entre IC e FFA é mediada por vários mecanismos, incluindo aumento e sobrecarga da pressão atrial, condução miocárdica alterada, expressão gênica mal adaptativa e remodelação estrutural.^23 , 27 - 29^ Ambas as condições complicam uma à outra e aplicam um efeito prejudicial significativo à saúde cardiovascular, sendo atualmente um alvo importante de pesquisa.

Um estudo recente mostrou que o aumento atrial ( *hazard ratio* : 1,53; intervalo de confiança de 95%: 1,27 a 1,85) e a disfunção sistólica ( *hazard ratio* : 1,80; intervalo de confiança de 95%: 1,01 a 3,26) se manifestaram com mais frequência entre pacientes com FA,^30^ reforçando nossos dados sobre o valor independente do aumento do AE e pior função do VE na FFA prevalente. Há uma justificativa plausível em que a remodelação atrial adversa interfere na atividade elétrica cardíaca, manifestando-se como alterações eletrocardiográficas como BRE, que pode ser um precursor da FFA, conforme bem descrito no contexto de IC,^31 - 33^ o que está ligado ao nosso achado eletrocardiográfico como um fator de risco para FFA prevalente.

A identificação prévia de fatores de risco para FFA permitiu a elaboração de escores de risco para prever o desenvolvimento dessa arritmia com bom desempenho (CHARGE-AF:^14^ estatística C 0,765 e EHR:^15^ estatística C 0,777). Em nosso estudo, a maioria dos indivíduos com ritmo sinusal foi categorizada como tendo baixo risco de desenvolver FFA em 5 anos, independentemente do escore usado. A proporção de alto risco para FFA em nossa amostra foi semelhante à descrita em um estudo incluindo 88.572 indivíduos com mais de 65 anos de idade de uma coorte baseada na população.^34^ Além disso, observamos que o EHR rebaixou com mais frequência o risco de indivíduos previamente classificados como risco moderado ou alto pelo CHARGE-AF. Essa tendência também foi observada em um estudo envolvendo mais de 4 milhões de indivíduos, onde a discriminação de FA do EHR foi ligeiramente maior em comparação ao CHARGE-AF.^35^

### Limitações

O presente estudo tem algumas limitações. Por ser uma análise transversal, não pudemos estabelecer causalidade e relações temporais. Uma grande proporção de participantes teve FFA definida por diagnóstico autorrelatado (68%); no entanto, restringir o diagnóstico de FFA ao registro eletrocardiográfico subestimaria o reconhecimento da FFA paroxística. Ademais, 36% da população foi excluída devido à falta de dados sobre o ritmo cardíaco basal ou ecocardiograma, demonstrando poucas diferenças nas características clínicas em comparação aos participantes estudados, o que torna improvável que essa limitação tenha afetado os resultados do presente estudo de forma importante. Finalmente, devemos também reconhecer que os modelos de previsão para FFA incidente não foram validados para a população brasileira.

## Conclusão

A presença de FFA foi associada ao histórico de IC, BRE, dilatação do AE e menor função sistólica do VE neste país de renda média. Além disso, 12% a 13% dos pacientes em ritmo sinusal estavam em alto risco de desenvolver FFA. A vigilância clínica e o monitoramento dos parâmetros de eletrocardiográficos e ecocardiográficos podem ajudar na identificação precoce de indivíduos com maior risco de FFA, permitindo intervenções precoces e provavelmente minimizando as complicações associadas à FFA.
