# Supplementary material for: Características Associadas à Fibrilação Atrial Prevalente e Perfil de Risco para Fibrilação Atrial Incidente em uma População Idosa do ELSA-Brasil
Source: Arq Bras Cardiol. 2025 Jan 30;122(1):e20240487. [Article in Portuguese] doi: 10.36660/abc.20240487 (PMC11805529; doi:10.36660/abc.20240487)
Supplement: Supplementary file 1 [file 0066-782X-abc-122-1-e20240487-suppl01.pdf]

SUPPLEMENTARY MATERIAL

Table 1 - Comparison of the participants 60 years and over of the ELSA-Study included in the study versus those excluded in this analysis

	**All participants (n=3,263)**	**Included (n=2,088)**	**Excluded (n=1,175)**	***p*-value**
Age - years	65.1 ± 4.1	65.1 ± 4.1	65.3 ± 4.2	0.059
Women - n (%)	1,735 (53%)	1,111 (53)	624 (53)	0.95
Race white - n (%)	1,796 (55.5)	1,179 (56.9)	617 (53.1)	***0.03***
Weight - kg	72.28 ± 14.19	72.04 ± 14.01	72.72 ± 14.51	0.19
Body mass index - kg/m^2	27.28 ± 4.61	27.09 ± 4.49	27.63 ± 4.81	***0.001***
Obesity - n (%)	777 (23.8)	468 (22.4)	309 (26.3)	***0.01***
Hypertension - n (%)	1,893 (58.1)	1,211 (58.1)	682 (58.2)	0.94
Systolic blood pressure - mmHg	129.1 ± 19.2	129.1 ± 19.02	129.1 ± 19.59	0.97
Diastolic blood pressure - mmHg	76.31 ± 10.61	76.51 ± 10.71	75.95 ± 10.44	0.14
Diabetes Mellitus - n (%)	919 (28.1)	549 (26.3)	370 (31.4)	***0.002***
Heart Failure - n (%)	125 (3.8)	77 (3.69)	48 (4.10)	0.56
Acute myocardial infarction - n (%)	138 (4.2)	90 (4.3)	48 (4.1)	0.76
Stroke - n (%)	87 (2.67)	53 (2.54)	34 (2.89)	0.54
Active smoking - n (%)	309 (9.47)	171 (8.19)	(138 11.7)	***0.003***
Dyslipidemia - n (%)	2,808 (86.6)	1,805 (88.8)	1,003 (86.3)	0.68
Chronic kidney disease - n (%)	277 (8.49)	157 (7.5)	120 (10.2)	***0.008***
Hypothyroidism - n (%)	352 (10.8)	219 (10.5)	133 (11.4)	0.41
CHADsVASc	2.09 ± 1.22	2.06 ± 1.20	2.13 ± 1.25	0.13
ASCVD 2013 (%)	11.9 (6.5 - 20.2)	11.4 (6.2 - 19.8)	12.6 (7 - 20.8)	***0.010***

Numbers represent mean ± SD for continuous variables and n (%) for categorical variables; ASCVD - Atherosclerotic Cardiovascular Disease Risk Estimate in 10-year

Table 2 - Baseline clinical and demographic characteristics of the studied sample of participants 60 years and over of the ELSA-Study according to the way of diagnosis AFF

	**Non-AFF (n=2,000)**	**Atrial fibrillation or flutter**			***p*-value**
		**By ECG recording (n=24)**	**By self-report only (n=64)**	**All AFF cases (n=88)**	
Age – Years	65 ± 4.0	67.8 ± 4.7	66.3 ± 4.3	66.8 ± 4.4	***0.006***
Women - n (%)	1.068 (53.4)	7 (29.1)	36 (56.2)	43 (50)	***0.018***
White race - n (%)	1.125 (56.7)	18 (75)	36 (56)	54 (61.3)	0.07
Weight – kg	72 ± 13.97	78 ± 18.3	70.8 ± 13.9	72.84 ± 14.9	0.11
Body mass index - kg/m^2	27.1 ± 4.49	27.6 ± 4.5	26.6 ± 4.41	26.9 ± 4.44	0.57
Obesity - n (%)	447 (22.35)	7 (29.1)	14 (21.9)	21 (23.86)	0.42
Hypertension - n (%)	1155 (57.8)	16 (66.6)	40 (62.5)	56 (63.63)	0.38
Systolic blood pressure - mmHg	129.2 ± 19.14	127.9 ±13.7	127.4 ±16.8	127.5 ± 15.9	0.65
Diastolic blood pressure - mmHg	76.57 ± 10.74	81.2 ± 9.9	73 ± 9.2	75.26 ± 10.0	***0.03***
Diabetes Mellitus - n (%)	520 (26.01)	12 (50)	17 (26.5)	29 (32.95)	***0.008***
Heart Failure - n (%)	63 (3.15)	4 (16.7)	10 (15.8)	14 (16.09)	***<0.001***
Acute myocardial infarction - n (%)	77 (3.85)	0 (0)	13 (20.3)	13 (14.77)	0.32
Stroke - n (%)	49 (2.45)	1 (4.17)	3 (4.69)	4 (4.55)	0.59
Active smoking - n (%)	161 (8.05)	6 (16.6)	6 (9.4)	10 (11.36)	0.11
Dyslipidemia - n (%)	1727 (86.7)	19 (79.2)	59 (92.2)	78 (88.6)	0.27
Chronic kidney disease - n (%)	146 (7.3)	4 (16.6)	7 (10.9)	11 (12.5)	0.08
Hypothyroidism n (%)	207 (10.4)	2 (8.3)	10 (15.6)	12 (13.6)	0.74
Charge-AF score					***<0.001***
Low risk - n (%)	1.260 (63)	5 (20.8)	33 (51.6)	38 (43.2)	
Intermediate risk - n (%)	500 (25)	8 (33.3)	13 (20.3)	21 (23.9)	
High risk - n (%)	240 (12)	11 (45.8)	18 (28.1)	29 (32.9)	
EHR score					***<0.001***
Low risk - n (%)	1.340 (67)	5 (20.8)	39 (60.9)	44 (50)	
Intermediate risk - n (%)	400 (20)	8 (33.3)	11 (17.2)	19 (21.59)	
High risk - n (%)	260 (13)	11 (45.8)	14 (21.8)	25 (28.4)	
CHADsVASc	2.04 ± 1.20	2.62 ± 1.6	2.51 ± 1.12	2.54 ± 1.27	0.09
ASCVD 2013 - %	11.4 (6 - 19.7)	21.5 (13.7 - 31.3)	11.1 (7.7 - 19.9)	13.8 (8.4- 22)	***<0.001***

Numbers represent mean ± SD for continuous variables and n (%) for categorical variables; ASCVD - Atherosclerotic Cardiovascular Disease.
The p-values are presented for comparison between the group without AFF (n=2,000) and the cases of AFF diagnosed by ECG (n=24).

Table 3 - Baseline electrocardiographic parameters of the studied sample of participants 60 years and over of the ELSA-Study according to the way of diagnosis AFF

	Non-AFF (n=2,000)	Atrial fibrillation or flutter			*p*-value
		By ECG recording (n=24)	By self-report only (n=64)	All AFF cases (n=88)	
Major Q wave abnormalities - n (%)	84 (4.2)	1 (4.1)	2 (3.1)	3 (3.4)	0.99
Complete right bundle branch block - n (%)	76 (3.8)	1 (4.1)	3 (4.7)	4 (4.6)	0.93
Complete left bundle branch block - n (%)	18 (0.91)	0	4 (6.3)	4 (4.6)	0.63
LVH with ST-T changes - n (%)	14 (0.71)	0	0	0 (0)	0.68
Long QT - n (%)	39 (1.96)	1 (4.1)	6 (9.3)	7 (7.95)	0.44
Left axis deviation - n (%)	119 (6)	2 (8.3)	4 (6.3	6 (6.9)	0.63
Right axis deviation - n (%)	1 (0.05)	0	0	0	0.91
Ventricular extrasystoles - n (%)	8 (0.40)	0	1 (1.6)	1 (1.15)	0.75
Low QRS voltage - n (%)	37 (1.86)	2 (8.3)	1 (1.59)	3 (3.45)	***0.02***

LVH - left ventricular hypertrophy.
The p-values are presented for comparison between the group without AFF (n=2,000) and the cases of AFF diagnosed by ECG (n=24).

Table 4 - Baseline echocardiographic parameters of the studied sample of participants 60 years and over of the ELSA-Study according to the way of diagnosis AFF

	Non-AFF (n=2,000)	Atrial fibrillation or flutter			*p*-value
		By ECG recording (n=24)	By self-report only (n=64)	All AFF cases (n=88)	
Left atrial diameter - cm	3.60 ± 0.4	4.43 ± 0.9	3.69 ± 0.5	3.91 ± 0.73	***<0.001***
Left atrial volume index - mL/m^2	27 ± 7.92	44.40 ± 19.4	28.6 ± 8.97	33.3 ± 14.8	***<0.001***
LV end-diastolic diameter - cm	4.47 ± 0.4	4.78 ± 0.69	4.57 ± 0.54	4.63 ± 0.6	0.05
LV end-systolic diameter - cm	2.8 ± 0.45	3.32 ± 0.85	2.97 ± 0.59	3.07 ± 0.7	***0.01***
Septal thickness - cm	1.03 ± 0.2	1.04 ± 0.16	1.01 ± 0.18	1.02 ± 0.18	0.74
Posterior wall thickness - cm	0.92 ± 0.1	0.98 ± 0.16	0.94 ± 0.12	0.95 ± 0.13	0.11
LV ejection fraction - %	67.4 ± 6.4	59.9 ± 10.3	65.19 ± 8.18	63.6 ± 9.1	***0.003***
LV mass/BSA - g/m^2	84.8 ± 22	92.13 ± 25.3	88.67 ± 21.7	89.6 ± 22.6	0.19
Moderate to severe left valve disease - n (%)	36 (1.8)	3 (12.5)	3 (4.69)	6 (6.8)	***<0.001***
LV geometric patterns - n (%)					0.29
Normal	886 (49.8)	8 (36.3)	29 (50.9)	37 (46.7)	
Concentric remodeling	632 (35.5)	9 (40.9)	15 (26.3)	24 (30.4)	
Concentric hypertrophy	145 (8.2)	4 (18.18)	8 (14)	12 (15.2)	
Eccentric hypertrophy	116 (6.5)	1 (4.55)	5 (8.77)	6 (7.6)	
E/e' ratio	8.7 ± 2.5	8.48 ± 2.28	8.9 ± 2.38	8.8 ± 2.3	0.67

Numbers represent n (%). LV - left ventricular; BSA - body surface area.
The p-values are presented for comparison between the group without AFF (n=2,000) and the cases of AFF diagnosed by ECG (n=24).
