# Supplementary material for: Características Associadas à Fibrilação Atrial Prevalente e Perfil de Risco para Fibrilação Atrial Incidente em uma População Idosa do ELSA-Brasil
Source: Arq Bras Cardiol. 2025 Jan 30;122(1):e20240487. [Article in Portuguese] doi: 10.36660/abc.20240487 (PMC11805529; doi:10.36660/abc.20240487)
Supplement: Translation

# Translation: Characteristics Associated with Prevalent Atrial Fibrillation and Risk Profile for Incident Atrial Fibrillation an Elderly Population from ELSA-Brasil

**DOI:** 10.36660/abc.20240487i

**Authors:** Bernardo Boccalon, Murilo Foppa, Luisa C.C Brant, Marcelo M. Pinto-Filho, Antonio L. Ribeiro, Bruce B. Duncan, Angela Barreto Santiago Santos

**Affiliations:** 1 Brazil Universidade Federal do Rio Grande do Sul, Porto Alegre, RS – Brazil; 2 Brazil Hospital de Clinicas de Porto Alegre, Porto Alegre, RS – Brazil; 3 Brazil Universidade Federal de Minas Gerais, Belo Horizonte, MG – Brazil

**Keywords:** Atrial Fibrillation, Aged, Risk Factors, Epidemiology

## Abstract

**Background:**

Atrial fibrillation (AF) is an arrhythmia causing significant symptoms and raising the risk of complications.

**Objectives:**

To evaluate the association of clinical, electrocardiographic, and echocardiographic parameters with prevalent atrial fibrillation or flutter (AFF) and assess the risk profile for incident AFF using the AF prediction scores CHARGE-AF and EHR in an elderly population from a developing country.

**Methods:**

We included all participants in ELSA-Brasil aged 60 and over whose diagnosis of AFF could be defined through self-report or electrocardiogram and who had echocardiography performed at the study’s baseline. For statistical analysis, results with p values < 0.05 were considered statistically significant.

**Results:**

Among the 2,088 participants (65 ± 4.1 years; 53% women), 88 (4.2%) had AFF. Those with AFF were older and had higher rates of heart failure (HF), previous myocardial infarction, left bundle branch block (LBBB), prolonged QT interval, supraventricular extrasystoles, and sinus bradycardia. They also had larger left atrial and left ventricular dimensions, and lower left ventricular ejection fraction (LVEF). Multivariable analysis showed that HF, LBBB, larger left atrium, and lower LVEF were independently associated with AFF. The 5-year risk for incident AFF was low (< 2.5%) in 63% and high (> 5%) in 12% of individuals according to the CHARGE-AF score, and low in 67% and high in 13% according to the EHR.

**Conclusion:**

AFF was found in 4.2% of this older Brazilian cohort. AFF was linked to HF history, LBBB, left atrial dilation, and reduced LVEF. Additionally, 12% to 13% of patients in sinus rhythm were at high risk for AFF. Monitoring clinical, electrocardiographic, and echocardiographic parameters can aid in early identification of high-risk individuals.

## Introduction

Atrial fibrillation (AF) is the most common sustained cardiac arrhythmia, and it can lead to limiting symptoms and increased risk of stroke, myocardial infarction (MI), heart failure (HF), and all-cause mortality.^1 - 3^The prevalence of AF varies among different populations, reaching 1% to 2% of the general population, and increases with age, particularly in individuals over 60 years old.^4^However, the limited availability of data on the prevalence of AF in low- and middle-income countries presents a significant challenge to the global efforts of AF knowledge.^5^

Atrial fibrillation or flutter (AFF) is a growing problem in Brazil due to its epidemiological transition, with the accelerated aging of our population and the increase in cardiovascular diseases. In the ELSA-Brasil, a large multicenter cohort aged 35 to 74 in Brazil, the prevalence of AFF was 2.5%, progressively increasing with aging (1.2% for patients < 45 years and 5.4% for those > 64 years).^6^Another Brazilian study that included 1,524 individuals aged 65 or over found a 2.4% prevalence of this arrhythmia.^7^

The presence of risk factors over decades may justify the increase in AFF incidence with aging, making the elderly more susceptible to the development of arrhythmia.^8^Furthermore, in the elderly, AFF is associated with a significantly higher risk of complications, as well as a higher chance of progressing to permanent AFF, compared to young people.^9^Considering that approximately 40% of individuals with AFF are clinically asymptomatic, the diagnosis of this arrhythmia may only occur after the development of its consequences.^10^

Identifying risk factors associated with AFF, as well as individuals who have higher risk scores for developing this arrhythmia, can help to monitor new cases or delay disease progression, allowing early treatment and reduction of complications. Thus, we sought to evaluate the association of clinical, electrocardiographic (ECG), and echocardiographic parameters with prevalent AFF in adults aged 60 years or more in a large Brazilian cohort and to describe the risk profile of new cases of AFF in this population.

## Methods

### Study population

ELSA-Brasil is a prospective cohort study designed to investigate cardiovascular disease and diabetes in 15,105 men and women, civil servants from universities or research institutions in six cities in Brazil. All active or retired employees aged 35 to 74 years were eligible for the study. We included all participants aged 60 years or older whose AFF diagnosis could be assessed at baseline and who had echocardiography at the study baseline. From the initial sample with participants ≥ 60 years (n = 3,263), 400 participants were excluded due to unavailable data about AFF (8 due to invalid ECG and 392 due to missing information with respect to previous AFF). Of the remaining 2,863 participants, 2,088 had echocardiographic images available for analysis ( Figure 1 ).

**Figure 1:** – Study flowchart.

The details of the study, including design, eligibility criteria, sources, recruitment methods, and measurements obtained have been described elsewhere.^11 - 13^The study protocol was approved by the Institutional Review Committee of each participating center, and written informed consent was provided by all participants. The present investigation was a cross-sectional study of the ELSA-Brasil during the first visit (August 2008 to December 2010).

### Diagnosis of atrial fibrillation or flutter

The present study defined the diagnosis of AFF at baseline if the participant (a) had an ECG recording with AFF in the ELSA-Brazil at baseline assessment (2008 to 2010) (n = 24) or (b) indicated that they had an AF diagnosis at an age younger than that of ELSA-Brasil enrollment by the questionnaire applied 4 years after enrollment (n = 64). Between 2012 and 2014, participants were invited to undergo onsite reassessment, which included new questionnaires. In this reassessment, they were asked the following question: “Did a physician ever say that you have/had atrial fibrillation?” Participants who answered “yes” to that question were asked, “How old were you the first time a physician told you that you have/had atrial fibrillation?”^6^

### Clinical variables

The choice of clinical variables was based on the variables included in the CHARGE-AF^14^and EHR^15^risk estimation formula. Demographic and clinical variables were obtained from the baseline visit according to standardized protocols. Hypertension was defined as systolic blood pressure ≥ 140 mmHg, diastolic blood pressure ≥ 90 mmHg measured at study clinic visit, or treatment with antihypertensive medication during the last 2 weeks. Participants were classified as having diabetes if they reported a previous diagnosis of diabetes, were taking medication for diabetes, or presented one of the following results in the laboratory tests: fasting blood glucose ≥ 126 mg/dl, 2-hour blood glucose ≥ 200 mg/dl, or HbA1C ≥ 6.5%. Dyslipidemia was defined as the use of lipid-lowering medication or any of the following results: LDL cholesterol level ≥ 130 mg/dl, total cholesterol > 200 mg/dl, HDL-C < 40 mg/dl in men or < 50 mg/dl in women, or triglycerides > 150 mg/dl. Glomerular filtration rate (GFR) was estimated using the Chronic Kidney Disease Epidemiology Collaboration (CKD-EPI) formula and defined as chronic kidney disease when GFR <60 ml/min.

Previous medical conditions, including the diagnosis of HF, MI, and stroke were obtained from study standardized interviews and questionnaires. Participants who declared having smoked at least 100 cigarettes throughout their lives and who continued to smoke were considered active smokers.^12 , 13^Race classification was based on self-declared reporting using categories of the Brazilian Demographic Census conducted by the Brazilian Institute of Geography and Statistics.^16^

Furthermore, 4 risk scores were calculated aiming to estimate the risk of embolic events (CHADsVASc), cardiovascular risk (Atherosclerotic Cardiovascular Disease [ASCVD]), and the 5-year incidence of AFF (EHR and CHARGE-AF) and, subsequently, to characterize our population by identifying the most seriously ill.

### Electrocardiographic variables

ECG variables were obtained from a baseline visit. The procedure for acquiring and reading ECGs has been detailed in a previous publication^11^and includes established quality assurance procedures. The ECGs were performed at each investigation center using the Burdick Atria 6100 device, at a calibration of 10 mm/mV and 25 mm/second speed. The exams were transmitted to the reading center electronically and were stored in a digital database for subsequent automated reading by the Glasgow System^17^and coding by the Minnesota Code.^18^All ECGs with AFF were verified manually by a physician.

### Echocardiographic analyses

All echocardiographic images were performed by trained echocardiographers at the first visit, using identical equipment (Aplio XG, Toshiba Corporation, Toshigi, Japan), with a 2.5 MHz sectoral transducer. Sequences of 3 cardiac cycles were selected in each echocardiographic window, recorded in digital format, and transferred to the echocardiographic reading center of ELSA-Brasil, along with a digital form with image quality and preliminary findings filled by the echocardiographer. All studies were analyzed blinded to other participant data on an offline dedicated workstation (ComPACS Review Station 10.5, Medimatic Solutions Srl, Italy). All measurements were made in triplicate following the recommendations of the American Society of Echocardiography^19^and included left ventricular (LV) diameters, LV wall thickness, LV mass, left ventricular ejection fraction (LVEF), and left atrial (LA) volume.

### AFF risk prediction models

We used 2 published risk prediction models for AFF: the CHARGE-AF and the electronic health record-AF (EHR), which estimate the 5-year cumulative risk of incident AFF according to 3 strata as low (< 2.5%), intermediate (2.5% to 5%), and high (> 5%) risk categories.^14 , 15^The predicted model CHARGE-AF for AFF considered factors such as age, race, height, weight, systolic and diastolic blood pressure, current smoking status, use of antihypertensive medication, diabetes, and history of acute MI and HF.^14^The EHR score was developed by analyzing data from 412,085 individuals. This model incorporated variables such as sex, age, race, smoking status, height, weight, diastolic blood pressure, hypertension, hyperlipidemia, HF, coronary heart disease, valve disease, history of stroke, peripheral arterial disease, chronic kidney disease, and hypothyroidism.^15^

### Ethics statement

This study was performed in line with the principles of the Declaration of Helsinki. Because it is a multicenter study, ELSA-Brasil’s research protocol was approved not only by the ethics committee of each institution, but also by the National Research Ethics Committee. Informed consent was obtained from all subjects involved in the study.

### Statistical analysis

All normally distributed data were displayed as mean and standard deviation (continuous data) or as count and proportion (categorical data). Continuous variables were compared using a 2-sided t test with unequal variance and categorical variables were compared using chi-squared tests. Continuous non-normal distributed data were described as median and interquartile range and analyzed using the Mann-Whitney U test. The Shapiro-Wilk test was used to assess data normality.

AFF's potential associated variables were analyzed using univariate logistic regression. A multivariable logistic model was used to identify clinically relevant, non-competing variables, using only parameters with statistical significance in univariate logistic regression. The risk prediction models for AFF were calculated and we used the Sankey diagram^20^to compare the overlapping of these two classifications. All tests were 2-sided and p values of < 0.05 were considered statistically significant. The statistical analyses were performed with STATA 14.0 (Stata Corp, College Station, TX, USA).

## Results

There were 88 participants (4.2%) with AFF among the 2,088 participants included in this study (65 ± 4.1 years, 53% female, and 57% self-declared white). The participants excluded from this analysis, due to missing information on AFF or echocardiography, were more obese with a higher prevalence of diabetes, chronic kidney disease, and active smoking, as shown in Supplementary Table 1 .

**Table 1:** – Baseline clinical and demographic characteristics of the studied sample of participants 60 years and over of the ELSA-Brasil Study

	All participants (n=2,088)	AFF (n=88)	Non-AFF (n=2,000)	p value
Age, years	65 ± 4.1	66.8 ± 4.4	65 ± 4.0	0.002
Women, n (%)	1,111 (53)	43 (50)	1,068 (53.4)	0.40
White race, n (%)	1,179 (56.9)	54 (61.3)	1,125 (56.7)	0.39
Weight, kg	72.04 ± 14.0	72.84 ± 14.97	72.01 ± 13.97	0.60
Body mass index, kg/m^2^	27.09 ± 4.49	26.9 ± 4.44	27.09 ± 4.49	0.69
Obesity, n (%)	468 (22.4)	21 (23.86)	447 (22.35)	0.73
Hypertension, n (%)	1,211 (58.1)	56 (63.63)	1155 (57.8)	0.28
Systolic blood pressure, mmHg	129.1 ± 19.02	127.5 ± 15.98	129.2 ± 19.14	0.35
Diastolic blood pressure, mmHg	76.51 ± 10.71	75.26 ± 10.03	76.57 ± 10.74	0.23
Diabetes mellitus, n (%)	549 (26.3)	29 (32.95)	520 (26.01)	0.14
Heart failure, n (%)	77 (3.69)	14 (16.09)	63 (3.15)	<0.001
Acute myocardial infarction, n (%)	90 (4.3)	13 (14.77)	77 (3.85)	<0.001
Stroke, n (%)	53 (2.54)	4 (4.55)	49 (2.45)	0.22
Active smoking, n (%)	171 (8.19)	10 (11.36)	161 (8.05)	0.26
Dyslipidemia, n (%)	1,805 (86.8)	78 (88.6)	1,727 (86.7)	0.60
Chronic kidney disease, n (%)	157 (7.5)	11 (12.5)	146 (7.3)	0.07
Hypothyroidism, n (%)	219 (10.5)	12 (13.6)	207 (10.4)	0.33
CHARGE-AF score				<0.001
Low risk, n (%)	1,298 (62.2)	38 (43.2)	1,260 (63)	
Intermediate risk, n (%)	521 (24.9)	21 (23.9)	500 (25)	
High risk, n (%)	269 (12.9)	29 (32.9)	240 (12)	
EHR score				<0.001
Low risk, n (%)	1,384 (66.3)	44 (50)	1,340 (67)	
Intermediate risk, n (%)	419 (20.1)	19 (21.6)	400 (20)	
High risk, n (%)	285 (13.6)	25 (28.4)	260 (13)	
CHADsVASc	2.06 ± 1.20	2.54 ± 1.27	2.04 ± 1.20	<0.001
ASCVD 2013, %	11.4 (6.2 - 19.8)	13.8 (8.44 - 22.3)	11.4 (6 - 19.7)	0.012

Numbers represent mean ± standard deviation or median and interquartile range for continuous variables and n (%) for categorical variables. AFF: atrial fibrillation or flutter; ASCVD: Atherosclerotic Cardiovascular Disease Risk Estimate in 10 years.

As shown in Table 1 , participants with AFF were older, and they had a worse cardiovascular risk profile. They presented a higher prevalence of previous HF and MI, a higher 10-year risk of cardiovascular events according to the ASCVD score, and a higher risk of embolic events related to AFF according to their calculated CHADsVASc score. We could not find differences related to sex, presence of hypertension, obesity, and history of stroke between groups. ECG abnormalities, such as complete left bundle branch block (LBBB), prolonged QT interval, supraventricular extrasystoles, and sinus bradycardia, were more prevalent in individuals with AFF ( Table 2 ). Echocardiographic parameters in participants with AFF demonstrated larger LA (linear and volumetric parameters) and LV dimensions, lower LVEF, and more prevalent moderate to severe left valve disease compared to non-AFF cases ( Table 3 ). In the supplemental material, we showed the analysis restricted to the 24 participants who had AFF in the baseline ECG (Supplementary Tables 2, 3, and 4).

**Table 2:** – Baseline electrocardiographic parameters of the studied sample of participants 60 years and over of the ELSA-Brasil Study

	All participants (n=2,064)	AFF (n=64)	Non-AFF (n=2,000)	p value
P wave duration, ms	110.05 ± 14.7	110.62 ± 16.5^*^	110.03 ± 14.71	0.79
Long PR interval,^#^n (%)	46 (2.22)	3 (3.45)^*^	43 (2.17)	0.42
Major Q wave abnormalities, n (%)	87 (4.2)	3 (3.4)	84 (4.2)	0.71
Complete right bundle branch block, n (%)	80 (3.8)	4 (4.6)	76 (3.8)	0.71
Complete left bundle branch block, n (%)	22 (1.06)	4 (4.6)	18 (0.91)	0.001
LVH with ST-T changes, n (%)	14 (0.68)	0 (0)	14 (0.71)	0.43
Long QT, n (%)	46 (2.2)	7 (7.95)	39 (1.96)	<0.001
Left axis deviation, n (%)	125 (6.04)	6 (6.9)	119 (6)	0.73
Right axis deviation, n (%)	1 (0.05)	0 (0)	1 (0.05)	0.83
Supraventricular extrasystoles, n (%)	27 (1.3)	4 (4.6)^*^	23 (1.16)	0.006
Ventricular extrasystoles, n (%)	9 (0.43)	1 (1.15)	8 (0.40)	0.30
Sinus bradycardia, n (%)	68 (3.27)	10 (11.36)^*^	58 (2.91)	<0.001
Low QRS voltage, n (%)	40 (1.93)	3 (3.45)	37 (1.86)	0.29

Numbers represent mean ± standard deviation and n (%) for categorical variables. *Data from participants who were in sinus rhythm during the baseline electrocardiogram, but were defined as having AFF by self-report. #Considered to be above 200 ms. AFF: atrial fibrillation or flutter; LVH: left ventricular hypertrophy.

**Table 3:** – Baseline echocardiographic parameters of the studied sample of participants 60 years and over of the ELSA-Brasil Study

	All participants (n=2,088)	AFF (n=88)	Non-AFF (n=2,000)	p value
Left atrial diameter, cm	3.61 ± 0.51	3.91 ± 0.73	3.60 ± 0.49	<0.001
Left atrial volume index, mL/m^2^	27.3 ± 8.44	33.3 ± 14.8	27 ± 7.92	<0.001
LV end-diastolic diameter, cm	4.48 ± 0.50	4.63 ± 0.6	4.47 ± 0.4	0.02
LV end-systolic diameter, cm	2.81 ± 0.47	3.07 ± 0.7	2.79 ± 0.45	0.001
Septal thickness, cm	1.03 ± 0.18	1.02 ± 0.18	1.03 ± 0.18	0.84
Posterior wall thickness, cm	0.92 ± 0.14	0.95 ± 0.13	0.92 ± 0.14	0.06
LV ejection fraction, %	67.3 ± 6.6	63.6 ± 9.1	67.4 ± 6.42	<0.001
LV mass/BSA, g/m^2^	85 ± 21.3	89.6 ± 22.6	84.8 ± 21.9	0.06
Moderate to severe left valve disease, n (%)	42 (2)	6 (6.8)	36 (1.8)	0.001
LV geometric patterns, n (%)				0.15
Normal	923 (49)	37 (46.7)	886 (49.8)	
Concentric remodeling	655 (35)	24 (30.4)	632 (35.5)	
Concentric hypertrophy	157 (9)	12 (15.2)	145 (8.2)	
Eccentric hypertrophy	122 (7)	6 (7.6)	116 (6.5)	
E/e' ratio	8.7 ± 2.5	8.8 ± 2.3	8.7 ± 2.5	0.72

Numbers represent mean ± standard deviation for continuous variables and n (%) for categorical variables. AFF: atrial fibrillation or flutter; BSA: body surface area; LV: left ventricular.

The odds ratios of each variable for the presence of AFF were summarized in Table 4 . In the multivariable logistic regression, history of HF, the presence of LBBB, left atrial volume index, and LVEF were independently associated with prevalent AFF.

**Table 4:** – Risk factors associated with the presence of atrial fibrillation or flutter at baseline of the ELSA-Brasil Study in the studied sample of participants 60 years and over

Variables	Univariable logistic regression			Multivariable logistic regression		
	Odds ratio	95% CI	p value	Odds ratio	95% CI	p value
Age, years	1.1	1.05-1.16	<0.001			
Women	0.87	0.56-1.34	0.53			
Body mass index, kg/m^2^	0.99	0.94-1.03	0.69			
Heart failure	5.9	3.15 - 11	<0.001	2.56	1.09-6.01	0.03
Previous myocardial infarction	4.32	2.3- 8.12	<0.001			
Hypertension	1.27	0.81-1.98	0.28			
Diabetes mellitus	1.39	0.88-2.20	0.14			
Stroke	1.89	0.66-5.37	0.22			
Complete left bundle branch block	5.26	1.74-15.9	0.003	4.97	1.23-20	0.024
Long QT	4.33	1.88-9.98	0.001			
Supraventricular extrasystoles	4.1	1.38-12.1	0.011			
Sinus bradycardia	4.27	2.1-8.69	<0.001			
LV end-diastolic diameter (cm)	1.75	1.16-2.65	0.007			
LV end-systolic diameter (cm)	2.38	1.65-3.42	<0.001			
Left atrial volume index (mL/m^2^)	1.06	1.04-1.08	<0.001	1.04	1.02-1.07	<0.001
LV mass/BSA (g/m^2^)	1.0	0.99-1.01	0.05			
LV ejection fraction (%)	0.94	0.90-0.96	<0.001	0.95	0.91-0.97	0.001
Moderate to severe left-sided valve disease	3.99	1.63-9.74	0.002			

AFF: atrial fibrillation or flutter; BSA: body surface area; CI: confidence interval; LV: left ventricular.

Both risk models showed that most of the 2,000 patients in sinus rhythm at the study baseline were at low risk for developing AFF. According to the CHARGE-AF, 63% of individuals were classified as low risk (< 2.5%), and 12% as high risk, with a risk of 5-year AFF greater than 5%. Similarly, according to the EHR, 67% of participants were classified as low risk, and 13% were considered high risk. In Figure 2 , we demonstrate the distribution of scores among individuals in sinus rhythm and the elevated overlap between the scores with 71% of them classified as similar risk in both scores. The most significant shift in the risk category occurred among those classified as intermediate risk according to the CHARGE-AF score, who were reclassified as low risk by the EHR score.

**Figure 2:** – Intersection between the AFF risk prediction profile in five years: CHARGE-AF and HER. AFF: atrial fibrillation or flutter.

## Discussion

In this study, we demonstrated that medical history of HF, LBBB, LA size, and LVEF were independently associated with prevalent AFF in an elderly Brazilian population. Additionally, we found that 12% to 13% of those in sinus rhythm were at high risk of developing AFF in the following 5 years, regardless of the risk score used.

Advanced age has consistently been identified as one of the main risk factors for AFF,^3 , 21 - 23^pointing to the potential role of cellular senescence in AF pathophysiology,^24^and recent changes in life expectancy can potentially increase the prevalence of this arrhythmia. Similar to a previous study in ELSA-Brasil,^6^the Rotterdam study (n = 6,808) revealed that the prevalence of AFF was 9% in individuals between 75 and 79 years old, increasing significantly to 17.8% in individuals aged 85 years and over.^25^In our study, we could not find that age was an independent factor for AFF prevalence, which may be explained by the fact that we restricted our sample to a limited range from 60 to 74 years. Moreover, the particularities of our sample, composed mainly of active public servants, can limit finding the association between AFF and other established factors for AFF. Stroke may have been underrepresented, due to the limitations imposed by this condition on participation in work activities and, consequently, in research. Furthermore, the epidemiological transition observed in the studied population, in which obesity was historically not a prevalent risk factor, may explain the absence of this risk factor in the elderly. Finally, the high prevalence of hypertension in both groups may have canceled the differences related to this specific situation.

We found that a history of HF was associated with prevalent AFF. Previously, Benjamin and co-authors demonstrated that the presence of HF increased by 6-fold the risk of developing AF in a long follow-up of the Framingham Heart Study (38 years).^26^This link between HF and AFF is mediated by various mechanisms, including atrial pressure overload and enlargement, altered myocardial conduction, maladaptive gene expression, and structural remodeling.^23 , 27 - 29^Both conditions complicate one another and apply a significant detrimental effect on cardiovascular health, being currently an important research target.

A recent study showed that atrial enlargement (hazard ratio 1.53; 95% confidence interval 1.27 to 1.85) and systolic dysfunction (hazard ratio 1.80; 95% confidence interval 1.01 to 3.26) manifested more frequently among patients with AF,^30^reinforcing our data about the independent value of LA enlargement and worse LV function in prevalent AFF. There is a plausible rationale where adverse atrial remodeling interferes with cardiac electrical activity, manifesting as ECG changes such as LBBB, which may be a precursor to AFF, which has been well described in the scenario of HF,^31 - 33^which is linked to our ECG finding as a risk factor for prevalent AFF.

The previous identification of AFF risk factors allowed the elaboration of risk scores to predict the development of this arrhythmia with good performance (CHARGE-AF:^14^C statistic 0.765 and EHR:^15^C statistic 0.777). In our study, most individuals with sinus rhythm were categorized as having a low risk of developing AFF within 5 years, regardless of the score used. The proportion of high risk for AFF in our sample was similar to that described in a study including 88,572 individuals over 65 years of age from a population-based cohort.^34^Furthermore, we observed that the EHR more frequently downgraded the risk of individuals previously classified as moderate or high risk by CHARGE-AF. This trend was also observed in a study involving over 4 million individuals, where the AF discrimination of EHR was slightly greater compared to CHARGE-AF.^35^

### Limitations

This study has some limitations. As this is a cross-sectional analysis, we cannot establish causality and temporal relationships. A large proportion of participants had AFF defined by self-reported diagnosis (68%); however, restricting AFF diagnosis to ECG recording would underestimate the recognition of paroxysmal AFF. Moreover, 36% of the population was excluded due to a lack of information on baseline heart rhythm or echocardiogram, demonstrating few differences in clinical characteristics compared to studied participants, thus making it unlikely that this limitation has affected important study results. Finally, we should also acknowledge that the prediction models for incident AFF were not validated for the Brazilian population.

## Conclusion

The presence of AFF was associated with HF history, LBBB, LA dilation, and lower LV systolic function in this middle-income country. Moreover, 12% to 13% of those in sinus rhythm were at high risk of developing AFF. Clinical surveillance and monitoring of ECG and echocardiography parameters may help the early identification of individuals with a higher risk of AFF, allowing early interventions and likely minimizing the complications associated with AFF.
